# Adolescent scoliosis and kyphosis treated with TLI (thoracolumbar lordotic intervention) technique in a brace. Mechanism and results

**DOI:** 10.1186/1748-7161-8-S1-P2

**Published:** 2013-06-03

**Authors:** PJM Van Loon, FB Thunnissen, M Roukens

**Affiliations:** 1 Orthopedium Delft, Delft, The Netherlands

## Background

Current braces in scoliosis only claim to stop progression[1]. Correctional forces in scoliosis and kyphosis push on bony structures. In etiology, lifestyle factors and exact knowledge on growth forces are important.

## Aim

To show that restoration of natural lordosis at the thoracolumbar junction, by applying controlled symmetrical lordotic forces, corrects scoliotic curves, thereby offering an alternative for present braces in scoliosis and kyphosis[2].

## Methods

Consecutive case control pilot study, with radiological results in coronal and sagittal curves, and scores for overall-satisfaction with TLI after one year of wearing. 91 consecutive children with scoliotic ( one curve >25º) and kyphotic spinal deformities during growth, wearing a lordotic brace at least a full year. Measurements of Cobb angles on AP and sagittal standing X-ray’s were compared at indication time, first-in brace day, and out of brace after a full year. A questionnaire was filled in with scores for satisfaction.

## Results

Mean age of start was late: 13.8 years (SD2). Menarche in girls (n= 46) was mean12.8 yrs (SD 1.2). In scoliosis (one curve at least 25º, N=38), the in-brace correction of the Cobb angles of thoracic and thoracolumbar curves and the pelvic obliquity was significant (p< 0.01 in all three). For the left lumbar curves p< 0.02. In the sagittal plane, even after a full year, a significant correction was seen in the thoracic and thoracolumbar curves. In kyphosis (pure or with maximal scoliosis of 25º, N=79), values for the thoracic curve (p< 0.01), the thoracolumbar curve (p< 0.01) ,the lumbar lordosis (p< 0,01) and the pelvic incidence (p< 0.01) , all in the sagittal plane, changed significantly in a paired t-test at one full year brace treatment in comparison with time of indication. Correction of high grade coronal curves (true bone remodelling) was still difficult and time consuming, whilst the end of growth is near. At all controls, active redressing was possible. Satisfied and very satisfied with the results were 84.6%. Choice for same treatment was 75.9%. Compliance was rated good in 60.2% and fair in 32%.

## Discussion

Sound etiologic factors were disclosed with consequences for brace techniques[3]. An identical technique of bracing (TLI) redirecting spines and muscular function tracts to original postures is invented. Good compliance and satisfaction seems part of the process.

## Conclusions

TLI is capable of reduction of all coronal and sagittal curves with a single symmetric force. Stepwise restoration of thoracolumbar lordosis, preventing anterior overload in compression, and derogating the spine gives rerouting of growth paths to more optimal postures.
